# Dangers of hyperoxia

**DOI:** 10.1186/s13054-021-03815-y

**Published:** 2021-12-19

**Authors:** Mervyn Singer, Paul J. Young, John G. Laffey, Pierre Asfar, Fabio Silvio Taccone, Markus B. Skrifvars, Christian S. Meyhoff, Peter Radermacher

**Affiliations:** 1 Bloomsbury Institute of Intensive Care Medicine, Division of Medicine, University College London, London, UK; 2 Medical Research Institute of New Zealand, and Intensive Care Unit, Wellington Hospital, Wellington, Wellington, New Zealand; 3 Australian and New Zealand Intensive Care Research Centre, Department of Critical Care Medicine, University of Melbourne, Melbourne, VIC Australia; 4 Department of Anaesthesia and Intensive Care Medicine, Galway University Hospitals, and School of Medicine, National University of Ireland, Galway, Ireland; 5 Département de Médecine Intensive - Réanimation Et Médecine Hyperbare, Centre Hospitalier Universitaire d’Angers, Angers, France; 6 Department of Intensive Care, Hôpital Erasme, Université Libre de Bruxelles, Bruxelles, Belgium; 7 Department of Emergency Care and Services, University of Helsinki and Helsinki University Hospital, Helsinki, Finland; 8 Department of Anaesthesia and Intensive Care, Bispebjerg and Frederiksberg Hospital, University of Copenhagen, Copenhagen, Denmark; 9 Institut für Anästhesiologische Pathophysiologie und Verfahrensentwicklung, Universitätsklinikum, Helmholtzstrasse 8-1, 89081 Ulm, Germany

**Keywords:** Hyperoxia, Hyperoxaemia, Reactive oxygen species, Reactive nitrogen species, ARDS, Sepsis, Trauma-and-haemorrhage, Traumatic brain injury, Subarachnoidal bleeding, Acute ischaemic stroke, Intracranial bleeding, Cardiopulmonary resuscitation, Myocardial infarction, Surgical site infection

## Abstract

Oxygen (O_2_) toxicity remains a concern, particularly to the lung. This is mainly related to excessive production of reactive oxygen species (ROS). *Supplemental O*_*2*_, i.e. inspiratory O_2_ concentrations (F_I_O_2_) > 0.21 may cause *hyperoxaemia* (i.e. arterial (a) PO_2_ > 100 mmHg) and, subsequently, *hyperoxia* (increased tissue O_2_ concentration), thereby enhancing ROS formation. Here, we review the pathophysiology of O_2_ toxicity and the potential harms of supplemental O_2_ in various ICU conditions. The current evidence base suggests that PaO_2_ > 300 mmHg (40 kPa) should be avoided, but it remains uncertain whether there is an “optimal level” which may vary for given clinical conditions. Since even moderately supra-physiological PaO_2_ may be associated with deleterious side effects, it seems advisable at present to titrate O_2_ to maintain PaO_2_ within the normal range, avoiding both hypoxaemia and excess hyperoxaemia.

**Supplementary Information:**

The online version contains supplementary material available at 10.1186/s13054-021-03815-y.

## Background

**Fig. 1:**
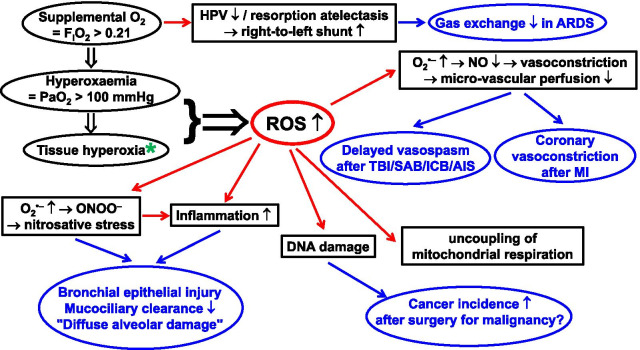
Potential harm of hyperoxia. AIS acute ischaemic stroke; MI myocardial infarction; ARDS acute respiratory distress syndrome; F_I_O_2_ fraction of inspired O_2_; HPV hypoxic pulmonary vasoconstriction; ICB intracranial bleeding; PaO_2_ arterial O_2_ partial pressure; NO nitric oxide; ONOO^‒^ peroxynitrite; O_2_^•‒^ superoxide anion; ROS reactive oxygen species; SAB subarachnoidal bleeding; TBI traumatic brain injury. * Note that while hyperoxia and hyperoxaemia are well defined as F_I_O_2_ > 0.21 and PaO_2_ > 100 mmHg, respectively, there is no general threshold for “tissue hyperoxia”, because the normal tissue PO_2_ depends on the macro- and microcirculatory perfusion and the respective metabolic activity. Nevertheless, it is noteworthy that PO_2_ levels as low as 0.3 – 0.7 mmHg suffice for correct functioning of the mitochondrial respiratory chain [17, 162]

Since its discovery [1–3], oxygen (O_2_) has been recognised as “friend and foe” [4]. It is vital for aerobic respiration within the mitochondria, yet mitochondrial respiration also forms reactive oxygen species (ROS) [5], production of which relates to O_2_ concentration [6–8]. Supplemental O_2_, i.e. inspiratory O_2_ concentrations (F_I_O_2_) > 0.21, may cause hyperoxaemia (arterial PO_2_ > 100 mmHg) and subsequently increased ROS formation [9–11]. This is particularly pronounced during ischaemia/reperfusion (I/R) and/or hypoxia/re-oxygenation [6–8]. ROS are as “Janus-headed” as O_2_: ROS are vital for host defence, and also toxic [12]. Consequently, O_2_ toxicity, especially pulmonary, is a matter of concern [13–15], and optimal dosing remains unclear in critical care. This review discusses potential harms of O_2_ in various underlying critical illnesses. Figure 1 summarises the possible dangers of hyperoxia, highlighting pathophysiological mechanisms and their impact on specific disease conditions. The most important clinical studies are listed in Table 1; “Additional file 1” shows the complete study list.

**Table 1:** Main features of the studies discussed in the text. ABG arterial blood gas; ACS acute coronary syndrome; AIS acute ischaemic stroke; AMI acute myocardial infarction; CI confidence interval; CPR cardiopulmonary resuscitation; ED emergency department; GCS Glasgow coma score; GOSE Glasgow outcome scale extended; ICU intensive care unit; IQR interquartile range; ICB intracranial bleeding; mo month; MV mechanical ventilation; OR odds ratio; RCT randomised controlled trial; ROSC return of spontaneous circulation; SAB subarachnoidal bleeding; SIRS systemic inflammatory response syndrome; SpO_2_ pulse oximetry haemoglobin O_2_ saturation; SOFA sequential organ failure assessment; SSI surgical site infection; STEMI ST segment elevation myocardial infarction; TBI traumatic brain injury; TWA time-weighted average

Study name	Design/sample size	Setting	Oxygenation parameter	Major findings	Ref. no.
IOTA	Meta-analysis/25 RCT, *n* = 16,037	General ICU	“Conservative” *vs.* “Liberal”, i.e. lower *vs.* higher target according to individual study design	Higher mortality risk (relative risk 1.21 [95%CI 1.0–1.43]) with “liberal” O_2_ strategy (median baseline SpO_2_ 96% [IQR 96–98%])	38
ICU-ROX	Multicentre RCT/*n* = 965	General ICU; MV	“Conservative” (lowest F_I_O_2_ possible keeping SpO_2_ between 91 and 97%) *vs.* “Usual” (no limit)	No difference in day 28 ventilator-free days and day 90/180 mortality	39
PROSPERO	Meta-analysis + Trial Sequential Analysis/36 RCT, * n* = 20,166	General ICU	“Lower” *vs.* “Higher”, i.e. lower vs. higher target according to individual study design	No difference in mortality or morbidity	42
O2-ICU	Multicentre RCT/*n* = 400	General ICU; expected ICU stay > 2 days; ≥ 2 SIRS criteria	Oxygenation target: PaO_2_ 8–12 *vs.* 14–18 kPa (≈ 60–90 vs. 105–135 mmHg)	No difference in SOFA score; *limitation:* PaO_2_ < target in “high-normal oxygenation” group	43
LOCO2	Multicentre RCT/*n* = 205	ARDS	“Conservative” (PaO_2_ 55–70 mmHg, SpO_2_ 88–92%) *vs.* “Liberal” (PaO_2_ 90–105 mmHg, SpO_2_ ≥ 96%) until day 7	Premature halt for higher mortality in “Conservative” group (day 28: 34.3 *vs.* 26.5%; day 90: 44.4 *vs.* 30.4%); *limitation:* > 50% patients had PaO_2_ > upper level	63
HOT-ICU	Multicentre RCT / *n* = 2,888	General ICU; acute hypoxemic respiratory failure	“Lower” (PaO_2_≈60 ± 7.5 mmHg) *vs.* “Higher” (PaO_2_≈90 ± 7.5 mmHg)	No difference in day 90 mortality	64
LUNG SAFE	Sub-study of multicentre, prospective, cohort study/* n* = 2,005	ARDS	Presence of day 1 “hyperoxemia” PaO_2_ > 100 mmHg), “sustained” (day 1 *and* day 2) or “excessive” O_2_ (F_I_O_2_ ≥ 0.6 + PaO_2_ > 100 mmHg)	30% hyperoxaemia day 1, 12% “sustained hyperoxaemia”, 20% “excessive O_2_”	65
IMPACT	Multicentre retrospective/*n* = 16,326	CPR; ABG within 24 h	PaO_2_ < 60 (“hypoxia”), 60–300 (“normoxia”), ≥ 300 mmHg (“hyperoxia”)	PaO_2_ ≥ 300 mmHg significantly higher mortality 63(CI:60–66)% *vs.* normoxia 45[CI43-48]%) vs. hypoxia (57[CI56-59]%)	68
HYPER2S	Multicentre RCT/*n* = 442	Septic shock within first 6 h; MV	F_I_O_2_ = 1.0 during first 24 h *vs.* “standard treatment”	Premature safety stop for higher mortality with “F_I_O_2_ = 1.0” (day 28: 43 *vs.* 35%, p = 0.12; day 90: 48 vs. 42%, p = 0.16); lower number of ventilator-free days, more serious adverse events despite lower SOFA at day 7	75
HYPER2S	Post hoc analysis of multicentre RCT/*n* = 393	Septic shock within first 6 h according to Sepsis-3; MV	F_I_O_2_ = 1.0 during first 24 h vs. “standard treatment”	Higher mortality with “F_I_O_2_ = 1.0” *and* lactate > 2 mmol/L (day 28: 57 *vs.* 44%); no effect lactate ≤ 2 mmol/L	76
ICU-ROX	Post hoc analysis of multicentre RCT/*n* = 251	Sepsis; MV	“Conservative” (lowest F_I_O_2_ possible keeping SpO_2_ between 91 and 97%) *vs.* “Usual” (no limit)	Mortality day 90 “Conservative” 36.2 *vs.* “Usual” 29.2% (p = 0.24); *“…point estimates of treatment effects consistently favoured usual O*_*2*_* therapy…”*	77
	Multicentre, retrospective/*n* = 1,116	TBI; MV	PaO_2_ < 10.0 kPa (≈ < 75 mmHg) or 10.0–13.3 kPa (≈ 75-100 mmHg) or PaO_2_ > 13.3 kPa (≈ > 100 mmHg)	PaO_2_ > 13.3 kPa no relationship to outcome	86
	Multicentre retrospective/*n* = 2,894	MV; 19% AIS, 32% SAB, 49% ICB	PaO_2_ < 60, 60–300 or ≥ 300 mmHg	PaO_2_ ≥ 300 mmHg in-hospital mortality 57 vs. 46/47% (p < 0.001)	87
	Multicentre retrospective/*n* = 432	SAB; MV	24 h TWA PaO_2_: “low”/“intermediate”/“high” (< 97.5/97.5–150/ > 150 mmHg)	TWA-PaO_2_: survivors 118(IQR90-155) *vs.* non-survivors 137(IQR104-167)mmHg (p < 001); multivariate analysis no relation between TWA-PaO_2_ and outcome	91
SO_2_S	Multicentre RCT/*n* = 7,635	AIS	Continuous (2-3L/min) *vs.* nocturnal nasal O_2_ *vs.* control	No difference in mortality and neurological outcome	92
	Multicentre retrospective/*n* = 24,148	TBI; MV	PaO_2_ 50 mmHg-increments; hyperoxia PaO_2_ > 300 mmHg	No relation PaO_2_ *vs.* mortality except for PaO_2_ < 60 mmHg *and* GCS > 12	93
	Multicentre retrospective/*n* = 3,699	TBI; MV	PaO_2_ < 60, 60–300 *vs.* PaO_2_ ≥ 300 mmHg	No relation PaO_2_ ≥ 300 mmHg *vs.* GOSE < 5 at 6 mo	95
	Single centre retrospective/*n* = 688	ED; MV, normoxia (PaO_2_ 60-120 mmHg) on day 1 ICU	Hypoxia/normoxia/hyperoxia PaO_2_ < 60, 60–120, > 120 mmHg	Hyperoxia present in 43%; mortality 29.7 *vs.* 19.4 (normoxia) and 13.2 (hypoxia) % (p = 0.021 vs. normoxia)	109
	Multicentre retrospective/*n* = 3,464	Polytrauma; ICU within 24 h	Patient-hours with SpO_2_ 90–96% (“normoxia”) *vs.* > 96% (“hyperoxia”); hyperoxia in 10%- F_I_O_2_ increments until d3 and d4-7	Increased risk of mortality with higher F_I_O_2_ during hyperoxia	114
IMPACT	Post hoc of multicentre retrospective/*n* = 4,459	CPR; ABG within 24 h	Highest PaO_2_ 24 h ICU	100 mmHg PaO_2_-increments 24% mortality risk increase (OR1.24[CI1.18–1.31])	121
	Multicentre prospective/*n* = 280	CPR; therapeutic hypothermia	PaO_2_ > 300 mmHg 1 or 6 h post-ROSC	3% (OR1.03[CI1.02–1.05]) risk increase in poor neurological outcome per 1 h hyperoxia duration	124
	Multicentre retrospective/*n* = 12,108	CPR; therapeutic hypothermia	PaO_2_ ≥ 300 mmHg within 24 h	PaO_2_ ≥ 300 mmHg mortality 59(CI56-61)% *vs.* 47(CI45-50% (60-300 mmHg)/58(CI57-58)% (< 60 mmHg)	125
FINNRESUSCI	Multicentre prospective/*n* = 409	CPR out-of-hospital	PaO_2_ < 75 (“low”), 75–150 (“middle”), 150–225 (“intermediate”), PaO_2_ > 225 mmHg (“high”)	No association between hyperoxia and neurological outcome	126
TTM	Post hoc analysis of multicentre RCT/*n* = 869	CPR out-of-hospital; therapeutic hypothermia	PaO_2_, TWA PaO_2_ 37 h post-ROSC; PaO_2_ > 40 kPa (≈PaO_2_ > 300 mmHg), 8 ≤ PaO_2_ ≤ 40 (≈60 ≤ PaO_2_ ≤ 300 mmHg), PaO_2_ < 8 kPa (≈PaO_2_ < 60 mmHg)	No association with 6-mo neurological outcome	129
	Meta-analysis/7 RCT, *n* = 429	CPR	“Higher” (“liberal”) *vs.* “lower” (“conservative”) O_2_ target	Mortality 50% liberal *vs.* 41% conservative, p = 0.04	130
ICU-ROX	Post hoc analysis of multicentre RCT/*n* = 166	“*Suspected hypoxic ischaemic encephalopathy*”; MV	“Conservative” (lowest F_I_O_2_ possible 91 ≤ SpO_2_ < 97%) *vs.* “Usual” (no limit)	Day 180: mortality 43% conservative vs. 59% “usual” (p = 0.15); “*unfavourable neurological outcome*” 55% conservative *vs.* 68% usual (p = 0.15)	134
DETO2X-SWEDEHEART	Multicentre RCT/*n* = 6629	AMI	6L/minO_2_ 6-12 h	No effect on 1-year outcome	138
Oxygen Therapy in Acute Coronary Syndromes	Multicentre crossover RCT/*n* = 40,872	ACS	6-8L/minO_2_ *vs.* SpO_2_ 90–95%	No effect on day 30-mortality	140
PROXI	Multicentre RCT/*n* = 1,386	Elective/acute laparotomy	F_I_O_2_ 0.8 *vs.* 0.3 until 2 h post-op	F_I_O_2_ 0.8 19.1% *vs.* F_I_O_2_ 0.3 20.1% SSI (p = 0.64)	143
Supplemental Oxygen in Colorectal Surgery	Single centre prospective/*n* = 5,749	Major intestinal surgery > 2 h	F_I_O_2_ = 0.8 *vs.* 0.3 every 2 weeks alternating intervention study	30d-SSI F_I_O_2_ = 0.8 10.8 *vs.* 11.0% (p = 0.85)	144
Intraoperative Inspiratory Oxygen Fraction and Postoperative Respiratory Complications	Multicentre retrospective/*n* = 79,322	General surgery	Quintiles F_I_O_2_ 0.31, 0.41, 0.52, 0.79	Dose-dependent association F_I_O_2_ *vs.* day 7 “Major respiratory complications composite” *and vs*. day 30-mortality	151
	WHO Meta-analysis/12 RCT, *n* = 5,976	General surgery	F_I_O_2_ 0.8 *vs.* 0.30–0.35	F_I_O_2_ = 0.8 reduces SSI risk *vs.* 0.30–0.35 (OR0.80[CI0.64–0.99], p = 0.043): only general anaesthesia with tracheal intubation	153
	Single centre RCT/*n* = 210	Open surgery for appendicitis	F_I_O_2_ = 0.8 *vs.* 0.30 until 2 h post-op	F_I_O_2_ = 0.8 SSI 5.6 *vs.*13.6% (p = 0.04); hospital stay 2.51 vs. 2.92 (p = 0.01)	156
Cochrane Perioperative Oxygen Review	Meta-analysis/10 RCT, *n* = 1,458	General surgery	“Higher” vs. “lower” F_I_O_2_	“Higher” vs. “lower” F_I_O_2_ “very low evidence” serious adverse event risk	157
	Meta-analysis/12 trials, *n* = 28,984	General ICU; MV	F_I_O_2_ “low” *vs.* “high” (as defined by authors)	F_I_O_2_ “high”; no impact on pneumonia, ARDS, MV duration; F_I_O_2_ ≥ 0.8 increased risk of: atelectasis	158

## Pathophysiology

Oxygen generally exists as di-atomic molecule (O_2_); its two atoms bond to each other through single bonds leaving two unpaired electrons. O_2_ performs its actions through these unpaired electrons which act as radicals. ROS are even more reactive molecules formed through oxygen’s electron receptivity (e.g. superoxide, peroxide, and hydroxyl anion).

Over 90% of O_2_ consumption is utilised by mitochondria, predominantly for ATP production (oxidative phosphorylation), but also for heat generation through uncoupling, and superoxide production. O_2_ is the terminal electron acceptor at Complex IV of the electron transport chain (ETC), being reduced to water in this process. For each mole of glucose metabolised, anaerobic respiration (glycolysis) generates only 2 ATP moles compared to approximately 28–30 from oxidative phosphorylation. In health, 1–3% of mitochondrial O_2_ consumption is used at the ETC Complexes I and III to generate superoxide, an important signalling molecule [16]. Superoxide is necessary for enzyme processes, e.g. oxidases (catalysing oxidation–reduction reactions) and oxygenases (incorporating oxygen into a substrate). Activated immune cells utilise O_2_ for extra-mitochondrial ROS production: NADPH oxidase generates superoxide (“respiratory burst”) for phagocytosis. Unless overwhelmed by ROS over-production, antioxidant capacity (e.g. superoxide dismutase, glutathione, thioredoxin) prevents oxidative damage to DNA, proteins and lipids, and subsequent cell death.

O_2_ also affects the inflammatory response. Experimental models and volunteer and patient studies demonstrate that hyperoxia (and hypoxia) can induce pro- and anti-inflammatory responses, with both protective and harmful sequelae [17]. Hyperbaric oxygen is used to aid wound healing and treat gas gangrene, but may cause neurotoxicity. Whether the response to hyperoxia relates to its degree and/or duration, specific cell types, background inflammation, or other factors remains uncertain; clearly, O_2_ toxicity can be induced de novo without underlying pathology, predominant organs being lung, brain, and eye.

Pulmonary toxicity was first described by Lorrain Smith: pure O_2_ at hyperbaric pressures caused inflammatory pneumonitis [18]. At atmospheric pressure pneumonitis was seen after days in non-human primates breathing 60–100% O_2_ [19–21]. After initially affecting the airways (tracheobronchitis) with reduced mucociliary clearance [22], the lung parenchyma becomes involved. In humans, this occurs especially when the inspiratory PO_2_ is significantly enhanced in a hyperbaric environment. Initial complaints are retrosternal chest pain, then coughing and dyspnoea as a pneumonitis develops with pulmonary oedema and diffuse radiological lung shadowing. In healthy volunteers breathing 98–100% O_2_, chest pain commenced after 14 h, coughing and dyspnoea between 30 and 74 h [22]. Due to nitrogen washout [23], there may also be atelectasis in lung regions with low ventilation/perfusion ratios [24].

Whether hyperbaric *vs.* normobaric O_2_ toxicity mechanisms and onset are similar is unclear. Pulmonary injury was accelerated by hyperbaric hyperoxia, but was less inflammatory in character and driven by a neurogenic component that could be blocked by inhibiting neuronal nitric oxide synthase or vagal nerve transection [25]. Possible synergistic effects on O_2_ toxicity of underlying lung pathology are poorly characterised, especially at the more moderate degrees of hyperoxia inflicted on patients. This is, however, well-recognised with bleomycin toxicity where mild hyperoxia may be damaging [26].

Neurotoxicity was described over a century ago [27]: 3Atm of O_2_ produced convulsions and death. Seizures or syncope occurred after 40 min at 4Atm O_2_, and within 5 min at 7Atm [28]. This was usually preceded by milder symptoms such as tunnel vision, tinnitus, twitching, confusion, and vertigo. The impact of high concentration normobaric O_2_ on neurotoxicity, however, is unclear.

Mitochondrial ROS production increases either with O_2_ deficit or excess, but particularly during excess O_2_ (hyperoxia). This can occur in sepsis and/or I/R injury, i.e. whole-body (e.g. resuscitation from cardiac arrest or major haemorrhage), or organ-specific (e.g. revascularisation after myocardial infarction or stroke). A similar injury may be induced by acute hypoxaemia followed by rapid correction (hypoxia/re-oxygenation-injury). The impact of reperfusion injury may be as severe as the ischaemic insult. Although preclinical and clinical studies are not consistent [29–31], reperfusion injury is generally exacerbated by hyperoxia. The hyperoxia effect may be exacerbated by acidification of the hypoxic tissues; the right-shifted oxyhaemoglobin dissociation curve of blood (re-)entering the hypoxic tissue augments O_2_ release, with a subsequent increase in superoxide production [31].

Teleologically, the body has not evolved to deal with high tissue O_2_ tensions. Tissues not metabolising adequately, e.g. due to toxins or switching off (“hibernating”) in response to hypoperfusion, reduce O_2_ utilisation. As a normal protective response, negative feedback signals reduce local blood flow by vasoconstriction to mitigate local build-up of O_2_ and subsequent toxicity. Acute hyperoxia thus induces vasoconstriction, reducing local blood flow [32], particularly in the cerebral and coronary vasculature [5–7]. This vasoconstriction is in part related to reduced release of nitric oxide (NO) from *S*-nitrosohaemoglobin binding [33]. Vasoconstriction has been shown in patients with and without coronary artery disease, where supplemental O_2_ reduced cardiac output and coronary sinus blood flow [34, 35]. Seizures associated with neurological O_2_ toxicity occur with paradoxical vasodilation during hyperbaric hyperoxia [6].

## General ICU patients

The Oxygen-ICU trial was the first major study to suggest clinically important harm from liberal O_2_ administration in a general ICU population [36]. This single-centre, RCT included 480 patients expected to stay in the ICU for at least 72 h. ICU mortality was 20.2% with conventional and 11.6% with conservative O_2_ therapy. Around two-thirds of patients included were mechanically ventilated at baseline, around a third had shock, and the illness acuity was relatively low. The difference was statistically significant although the study was stopped early after a non-preplanned interim analysis, and the magnitude of the reported treatment effect was larger than hypothesised [36]. Given the variety of mechanisms of death in ICU patients [37], such a high proportion of deaths in a heterogeneous population of ICU patients is unlikely to be attributable to the dose of O_2_ therapy used. However, the Oxygen-ICU trial [37] did highlight the need for further investigation.

Subsequently, the IOTA systematic review and meta-analysis [38] reported that conservative O_2_ use in acutely ill adults significantly reduced in-hospital mortality. Although these findings were concordant with the Oxygen-ICU trial [36], they provided only low certainty evidence: First, the Oxygen-ICU trial [36] contributed 32% of the weight to the mortality analysis. Second, predominant conditions were acute myocardial infarction and stroke, and a range of O_2_ regimens were tested so that the analysis provided only indirect evidence about the optimal O_2_ regimen for patients in the ICU. Third, the overall mortality treatment effect estimates were imprecise. Finally, an updated systematic review and meta-analysis found no evidence of benefit or harm comparing higher *vs.* lower oxygenation strategies in acutely ill adults [39].

The multicentre randomised ICU-ROX trial found that conservative O_2_ therapy did not significantly affect the primary end point of number of days alive and free from mechanical ventilation (ventilator-free days) compared with usual (liberal) O_2_ therapy [40]. Overall, 32.2% of conservative and 29.7% of usual O_2_ patients died in hospital. While these findings provide some reassurance to clinicians about the safety of the liberal O_2_ use that occurs in standard practice, they do not exclude clinically important effects of the O_2_ regimens tested on mortality risk. Indeed, based on the distribution of data, there is a 46% chance that conservative O_2_ therapy *increases* absolute mortality by more than 1.5% points, and a 19% chance that conservative O_2_ therapy *decreases* absolute mortality by more than 1.5% points [41, 42]. Finally, a recent RCT conducted in ICU patients fulfilling the systemic inflammatory response syndrome criteria, found no significant difference between high-normal and low-normal oxygenation targets for non-respiratory organ dysfunction over the first 14 days, or in Day-90 mortality [43]. Accordingly, the most appropriate dose of O_2_ to give to adult ICU patients remains uncertain.

## ARDS

Clinicians should titrate O_2_ therapy to avoid both hypoxaemia and hyperoxaemia. While the harmful effects of tissue hypoxia are clearly understood [44], over-correction leads to tissue hyperoxia which may also be deleterious. Hyperoxia injures the lung via ROS production, causing oxidant stress with pro-inflammatory and cytotoxic effects [35, 45, 46]. Pathophysiologic consequences include arterial vasoconstriction [35, 47–49], alveolar-capillary “leak” and even fibrogenesis [50, 51]. Clinicians use higher F_I_O_2_ than necessary to correct hypoxia in the critically ill [52], possibly to avoid (occult) tissue hypoxia [53, 54], to provide a “buffer” should rapid clinical deterioration occur, or because the consequences of hyperoxia are considered less severe. The lack of clearly defined targets for PaO_2_ and/or SaO_2_ is also an issue. The ARDS Network trials targeted a PaO_2_  of  55-80 mmHg [55], while the British Thoracic Society suggests a target SpO_2_ of  94–98% in acutely ill patients [56].

In ARDS, the potential for hyperoxia to impact outcomes is further complicated by the severity of gas exchange impairment. Specifically, extreme hyperoxaemia (i.e. PaO_2_ > 300 mmHg), associated with harm in other critically ill populations, is impossible to achieve in ARDS (see Table 1). However, moderate hyperoxaemia is possible and could be harmful as well [57]. Furthermore, high F_I_O_2_ can directly injure the lung [58], sensitise it to subsequent injury [59], adversely affect its innate immune response [60], and worsen ventilation-induced injury [61, 62]. It is therefore necessary to distinguish between hyperoxaemia and high F_I_O_2_ use when assessing the effects of hyperoxia on the lung.

The recent LOCO2 trial in ARDS was stopped early for futility and safety concerns regarding mesenteric ischaemia in the conservative O_2_ group. Moreover, 90-day mortality was significantly higher in patients receiving conservative O_2_ therapy [63]. The HOT-ICU trial studied ICU patients with acute hypoxaemic respiratory failure and found no difference in 90-day mortality between conservative and liberal PaO_2_ targets [64]. In the LUNG SAFE observational cohort study, both systemic hyperoxaemia and excess F_I_O_2_ use were prevalent, with frank hyperoxaemia (30% of patients) more prevalent than hypoxaemia in early ARDS [65]. Two-thirds of these patients received excess O_2_ therapy. Hyperoxaemia did not appear to be used as a “buffer” in unstable patients: frequency was similar in shocked patients. While a similar proportion of patients were hyperoxaemic on day-2, higher F_I_O_2_ use did decrease. Both hyperoxaemia and excess O_2_ use were mostly transient, although more sustained hyperoxaemia was seen. Reassuringly, no relationship was found between the degree and duration of hyperoxaemia, or excessive O_2_ use, and mortality in early ARDS.

While these findings contrast with findings in other ICU cohorts, a key differentiating factor is the reduced potential for extreme hyperoxia in ARDS patients. The potential for harm from hyperoxia appears to be related to the severity of hyperoxaemia [54, 61, 66, 67]; those with relatively preserved lung function are at greatest risk [68]. However, no dose–response relation was found between PaO_2_ and mortality [67]. Hence, paradoxically, patients with ARDS may be at less risk as they are unable to achieve extreme degrees of hyperoxia. A recent observational study suggested a U-shaped relationship between PaO_2_ and mortality in ARDS patients; patients with a time-weighted PaO_2_  of  93.8-105 mmHg had the lowest mortality risk [69]. Intriguingly, this range is near identical to the “*liberal*” target PaO_2_ range targeted in the LOCO2 [63]. Hence, much remains to be learned about optimal targeting of PaO_2_ in patients with ARDS.

## Sepsis and septic shock

Theoretically, hyperox(aem)ia might help septic patients due to its vasoconstrictor effect, counteracting hypotension [6–8], and to the antibacterial effects of O_2_ [70, 71]. However, hyperoxaemia did not affect cardiac output in septic patients [72]. The number of days with PaO_2_ > 120 mmHg was an independent risk factor for ventilator-associated pneumonia (VAP) [73]; however, these patients had other risk factors, e.g. more frequent use of proton pump inhibitors and sedatives, higher incidence of circulatory shock with prolonged and higher catecholamine infusion rates, and more red blood cell transfusion. In an observational study on VAP patients, the same group reported that hyperoxaemia did not affect mortality [74]. The HYPER2S RCT [75] compared standard therapy *vs*. 100% O_2_ over the first 24 h after diagnosing septic shock. Despite a significantly lower SOFA score at day 7, the trial was prematurely stopped due to higher, albeit not statistically significant mortality in the hyperoxia group at Day-28 and Day-90. The hyperoxia group had significantly more serious adverse events, including ICU-acquired weakness (p = 0.06). A post hoc analysis based on Sepsis-3 criteria found increased Day-28 mortality in patients with hyperlactataemia > 2 mmol/L (p = 0.054), but not with normal lactate levels [76]. The authors speculated that a hyperoxaemia-related increase in tissue O_2_ availability may have led to excess ROS production and, consequently, oxidative stress-related tissue damage.

The opposite hypothesis, i.e. attenuation of oxidative stress-induced tissue damage by reducing O_2_ exposure did not beneficially influence outcome in septic patients either. A post hoc analysis of the ICU-ROX trial [40] of the septic cohort showed no statistically significant difference with respect to ventilator-free days or Day-90 mortality for the “conservative” when compared to the “usual” oxygenation [77]. Point estimates of treatment effects even favoured the latter. Hence, it seems reasonable to avoid PaO_2_ > 100-120 mmHg due to the possible deleterious consequences of excess tissue O_2_ concentrations in the presence of sepsis-related impairments of cellular O_2_ extraction [78].

## Acute brain injury

Increasing F_I_O_2_ in acutely brain-injured patients, alongside other clinical interventions [79], can improve brain tissue PO_2_ (PbtO_2_) [80, 81]. The effects of normobaric hyperoxia are less significant in large hypoperfused brain regions [82], but highly relevant in small pericontusional areas [83]. Moreover, incremental F_I_O_2_ increased cerebral excitotoxicity in severe traumatic brain injury (TBI) [84]. The association of hyperoxia with outcome is even more controversial. After TBI, both hypoxaemia and hyperoxia were [85] or were not [86] independently associated with worse outcome. In two retrospective studies, including a mixed population of brain-injured patients, hyperoxaemia, defined as PaO_2_ > 300 mmHg [87] or > 120 mmHg [88], was associated with increased in-hospital mortality and poor neurological outcome, even after adjusting for confounders. Patients with subarachnoid haemorrhage exposed to higher PaO_2_ levels were also more likely to develop cerebral vasospasm [89, 90]; however, a retrospective analysis of patients needing mechanically ventilation did not find any relation between time-weighted PaO_2_ and outcome [91]. Studies in acute ischaemic stroke in general [92], and in a sub-group needing mechanical ventilation [93], found no association between outcome and PaO_2_ within the first 24 h. Even early hyperoxaemia (PaO_2_ > 300 mmHg) did not affect mortality in mechanically ventilated TBI patients, notwithstanding severity on admission [94, 95]. Finally, PaO_2_≈150-250 mmHg within the first 24 h post-TBI was associated with better long-term functional outcome after TBI [96]; however, the study excluded patient who died. Normobaric hyperoxia combined with intravenous thrombolysis was associated with more favourable neurological outcome than thrombolysis alone after ischaemic stroke [97].

Prospective studies have evaluated the effects of targeted hyperoxia after acute brain injury: Small studies in patients with acute ischaemic stroke not eligible for thrombolysis found either transient clinical improvement and smaller infarct size with high-flow O_2_ [98, 99] or no effect of normobaric hyperoxia [100]. In a small RCT in mechanically ventilated TBI patients, F_I_O_2_ = 0.8 (*vs.* 0.5) improved 6-month neurological outcome [101], but conclusions should be cautioned due to methodological concerns. Exposure to F_I_O_2_ = 0.7 or 0.4 for up to 14 days after TBI influenced neither markers of oxidative stress or inflammation nor neurological outcome [102]. Finally, the Normobaric-Oxygen-Therapy-in-Acute-Ischemic–Stroke-Trial (NCT00414726) was prematurely halted after inclusion of 85/240 patients because of higher mortality in the high-flow O_2_ group, although most deaths occurred following early withdrawal of life-support.

It remains open in acute brain injury, whether normoxaemia *vs.* targeted hyperoxaemia influences brain function and neurological recovery. Optimal PaO_2_ targets, study populations, and specific forms of brain injury are currently unknown.

## Trauma-and-haemorrhage

Supplemental O_2_ is used because increasing the amount of physically dissolved O_2_ during blood loss-related reductions in O_2_ transport is thought to faster repay a tissue O_2_ debt [103]. Despite its vasoconstrictor properties [6–8], ventilation with 100% O_2_ during experimental haemorrhage improved tissue PO_2_ [104] and attenuated organ dysfunction [105, 106].

However, PaO_2_ > 100 mmHg may enhance ROS formation [9–11], especially during I/R and/or hypoxia/re-oxygenation, e.g. resuscitation from trauma-and-haemorrhage [6–8].

A recent retrospective study in patients with prehospital emergency anaesthesia demonstrated that hyperoxia was present in most patients upon arrival in the hospital, however without relation to outcome [107]. Clinical data on the impact of hyperoxia on morbidity and mortality remain equivocal. No association was seen between mortality and PaO_2_ in the first 24 h (median Injury Severity Score ISS = 29) [108]. Another observational study noted that 44.5% of patients mechanically ventilated in the emergency department had hyperoxaemia, this cohort having a higher Day-28 mortality [109]. From a French trauma registry (median ISS = 16), univariate analysis showed that admission PaO_2_ > 150 mmHg coincided with a higher mortality, however, propensity score matching yielded the opposite result, namely supra-physiological PaO_2_ levels were associated with significantly lower mortality [110]. Lower Day-28 mortality and less nosocomial pneumonia were seen in patients early after blunt chest trauma [111]. An analysis of 864,340 trauma patients (median ISS = 9) investigated the impact of supplemental O_2_ in the ED; in all three patient categories predefined according to incremental SpO_2_, supplemental O_2_ was associated with a significantly higher ARDS incidence and mortality [112]. A retrospective analysis of patients with ISS ≥ 16 studied the impact of PaO_2_ ≥ 300 mmHg during resuscitation [113]; while prolonged ICU stay was seen in patients not intubated in the ED, no effect was seen in the sicker cohort of mechanically ventilated patients. Finally, a retrospective multicentre study of trauma patients found SpO_2_ > 96% over the first seven days was common place; the adjusted mortality risk was higher with greater F_I_O_2_ [114]. The currently recruiting “*Strategy-to-Avoid-Excessive-Oxygen-for-Critically-Ill-Trauma-Patients* (SAVE-O2)” (NCT04534959) will address any causality between hyperoxia and outcome.

Despite O_2_ supplementation being common practice in patients with pronounced blood loss, no optimal target for PaO_2_ is available.

## Cardiopulmonary resuscitation and myocardial infarction

During cardiac arrest, PbtO_2_ drops rapidly to levels close to zero [115]. With cardiopulmonary resuscitation (CPR) PbtO_2_ increases slowly, driven by the achieved cerebral perfusion pressure [116]. Guidelines recommend ventilation with 100%O_2_ even though no clinical study has compared this against lower F_I_O_2_ [117]. Observational data suggest an association between higher PaO_2_ during CPR and a higher likelihood of return of spontaneous circulation (ROSC), survival, and neurological outcome [118, 119]. After ROSC blood and brain PO_2_ levels increase; mostly, this appears inevitable as F_I_O_2_ titration is impossible during CPR [120]. Given the connection between hyperoxia and ROS formation, there has been great interest in assessing whether avoidance of hyperoxaemia in the post-arrest phase could mitigate brain injury. Results are conflicting, either showing an association between hyperoxia and poor outcome [68, 121–124], or not [125–129]. Smaller randomised trials and sub-group analysis from larger trials have also been performed [130]. Overall, the evidence suggests that lower rather than higher O_2_ targets are beneficial, even though any sweet spot for optimal PaO_2_ is unknown [131]. The COMACARE pilot trial compared different PaO_2_ targets and found no difference in two brain injury biomarkers [132, 133]. A sub-group analysis of the ICU-ROX study showed improved outcomes in restrictive compared to liberal O_2_ treated patients at risk of hypoxic brain injury [134]. Opposite findings were seen in a sub-group of the HOT-ICU trial [64]. Current guidelines recommend targeting strict normox(aem)ia. The evidence suggests a signal to harm and, importantly, no indication of benefit from extreme hyperox(aem)ia; thus, this should be avoided [135].

Supplemental O_2_ use has been standard practice for decades in acute myocardial infarction (AMI) [136]. Studies have nonetheless suggested side effects including coronary artery vasoconstriction [137]. Several large studies have shown either harm or lack of benefit from supplemental O_2_ use in patients without hypoxaemia [138, 139]. A large cluster randomised controlled trial of > 40,000 patients with acute coronary syndrome (including patients with AMI) found no benefit with supplemental O_2_ use overall, but evidence was inconclusive in patients with ST-elevation AMI [140]. Importantly, these trials included patients without hypoxaemia [138, 140]. Despite the lack of high-quality evidence, it appears prudent to avoid hypoxaemia (SaO_2_ < 90%) in AMI patients.

## Perioperative hyperoxia

Trials of intraoperative hyperoxia have mainly been performed in elective surgery to prevent surgical wound infection through increased tissue oxygenation [141, 142]. Initial enthusiasm was followed by larger trials with similar wound complication frequencies with F_i_O_2_ = 0.80 *vs.* 0.30 perioperatively [143, 144]. Concerns have been raised by shorter cancer-free survival in patients given F_i_O_2_ = 0.80 [145, 146]. A higher F_I_O_2_ is used to ensure adequate or, in some cases, supranormal end-organ oxygenation, although there is sparse evidence of benefit.

Both preoxygenation and high intraoperative F_I_O_2_ can cause resorption atelectasis [147], especially in patients with pulmonary comorbidity, as general anaesthesia itself reduces functional residual capacity and causes airway closure [148]. As ventilation-perfusion mismatch and shunt contribute to impaired oxygenation, use of F_I_O_2_ = 0.30–0.35 is therefore considered normal during general anaesthesia [149, 150]. F_I_O_2_ ≥ 0.80 caused significant atelectasis during preoxygenation, but this can be eliminated with a recruitment manoeuvre followed by 5-10cmH_2_O PEEP [14], which clearly is not common practice. Failure to correct such iatrogenic atelectasis may trigger the use of excessive perioperative F_I_O_2_. In a large observational study [151], high intraoperative F_I_O_2_ was dose-dependently associated with major pulmonary complications and mortality after adjustment for all relevant risk factors. This association has not yet been confirmed in RCTs [152].

Based on a sub-group analysis in a systematic review, the World Health Organization proposed using F_I_O_2_ = 0.80 in all intubated patients to prevent postoperative wound infections [153]. This engendered controversial discussion [154, 155]. Most of the evidence for risks and benefits of hyperoxia during emergency surgery arise from RCTs of 385 laparotomy procedures and 210 open appendicectomies [143, 156]. While wound infections were significantly reduced with F_I_O_2_ = 0.80 in the appendicectomy study, the frequencies of surgical site infections, serious adverse events and mortality did not differ in the laparotomy trial [156, 157].

Acute perioperative patients should be carefully treated with respect to their ongoing medical conditions; most current evidence suggests greatest safety with O_2_ titration to normoxaemia.

## Conclusions

Current evidence suggests that PaO_2_ > 300mmmHg should be avoided in most ICU patients. It remains uncertain whether there is a “sweet spot” PaO_2_ target, which may vary for given clinical conditions. Systematic reviews using *trial sequential analysis* to take into account high *vs.* low bias risk found no effect (including all patients [39]) or increased mortality (including only ICU patients [157]) from higher oxygenation targets. Certainty evidence was low with futility for a 15% relative mortality risk increase. The currently recruiting “*Mega-Randomised-Registry-Trial-Comparing-Conservative-vs.-Liberal-Oxygenation* (Mega-ROX trial)” (CTG1920-01) in 40,000 patients should provide any “ideal target PaO_2_”: The trial tests the hypothesis that conservative *vs.* liberal O_2_ targets reduce mortality by 1.5% points in mechanically ventilated, adult ICU patients, i.e. 1,500 lives saved for every 100,000 patients treated. Since both conservative *and* liberal O_2_ therapy may be best for certain patients, several parallel trials will evaluate pre-specified hypotheses in specific patient cohort patients accompanied by separate power calculations. For example, anticipating heterogeneity of treatment response, in septic patients or patients with acute brain pathologies (other than hypoxic brain injuries), the trial will test the opposite hypothesis that liberal (rather than conservative) O_2_ will reduce mortality. Finally, the trial design cannot exclude that for some patient sub-groups, a different window of O_2_ exposure is most suited.

So far, it appears prudent to target PaO_2_ values within the normal range, i.e. carefully titrating PaO_2_ to avoid both hypoxaemia and excess hyperoxaemia [158], particular as no clinically useful biomarker of O_2_ toxicity is available, and data on the effects of hyperoxia on markers of oxidative stress are equivocal [10, 159–161].

## Supplementary Information

**Additional file 1.** Main features of the studies discussed in the text. ABG arterial blood gas; ACS acute coronary syndrome; AIS acute ischaemic stroke; AMI acute myocardial infarction; CI confidence interval; CPR cardiopulmonary resuscitation; d day; ED emergency department; GCS Glasgow coma score; GOS Glasgow outcome scale; GOSE Glasgow outcome scale extended; ICU intensive care unit; IQR interquartile range; ICB intracranial bleeding; mo month; MV mechanical ventilation; OR odds ratio; PPI proton pump inhibitor; RBC red blood cell; RCT randomised controlled trial; ROSC return of spontaneous circulation; SAB subarachnoidal bleeding; SIRS systemic inflammatory response syndrome; SpO2 pulse oximetry haemoglobin O2 saturation; SOFA sequential organ failure assessment; SSI surgical site infection; STEMI ST segment elevation myocardial infarction; TBI traumatic brain injury; TWA time-weighted average; VAP ventilator-associated pneumonia.

## Data Availability

Not applicable.
