# Supplementary material for: Dangers of hyperoxia
Source: Crit Care. 2021 Dec 19;25:440. doi: 10.1186/s13054-021-03815-y (PMC8686263; doi:10.1186/s13054-021-03815-y)
Supplement: Supplementary file 1 — Additional file 1. Main features of the studies discussed in the text. ABG arterial blood gas; ACS acute coronary syndrome; AIS acute ischaemic stroke; AMI acute myocardial infarction; CI confidence interval; CPR cardiopulmonary resuscitation; d day; ED emergency department; GCS Glasgow coma score; GOS Glasgow outcome scale; GOSE Glasgow outcome scale extended; ICU intensive care unit; IQR interquartile range; ICB intracranial bleeding; mo month; MV mechanical ventilation; OR odds ratio; PPI proton pump inhibitor; RBC red blood cell; RCT randomised controlled trial; ROSC return of spontaneous circulation; SAB subarachnoidal bleeding; SIRS systemic inflammatory response syndrome; SpO2 pulse oximetry haemoglobin O2 saturation; SOFA sequential organ failure assessment; SSI surgical site infection; STEMI ST segment elevation myocardial infarction; TBI traumatic brain injury; TWA time-weighted average; VAP ventilator-associated pneumonia. [file 13054_2021_3815_MOESM1_ESM.docx]

**Additional file 1.** Main features of the studies discussed in the text. ABG arterial blood gas; ACS acute coronary syndrome; AIS acute ischaemic stroke; AMI acute myocardial infarction; CI confidence interval; CPR cardiopulmonary resuscitation; d day; ED emergency department; GCS Glasgow coma score; GOS Glasgow outcome scale; GOSE Glasgow outcome scale extended; ICU intensive care unit; IQR interquartile range; ICB intracranial bleeding; mo month; MV mechanical ventilation; OR odds ratio; PPI proton pump inhibitor; RBC red blood cell; RCT randomised controlled trial; ROSC return of spontaneous circulation; SAB subarachnoidal bleeding; SIRS systemic inflammatory response syndrome; SpO_2_ pulse oximetry haemoglobin O_2_ saturation; SOFA sequential organ failure assessment; SSI surgical site infection; STEMI ST segment elevation myocardial infarction; TBI traumatic brain injury; TWA time-weighted average; VAP ventilator-associated pneumonia.

| Study name | Design / Sample size | Setting | Oxygenation parameter | Major findings | Ref. no. |
| --- | --- | --- | --- | --- | --- |
| Oxygen-ICU | Single center RCT / n=434 | General ICU; expected ICU stay ≥3 days | "Conservative" (70≤PaO_2_≤100 mmHg or 94≤SpO_2_≤98 %) *vs.* "Conventional" (PaO_2_≤150mmHg or 97≤SpO_2_≤100 %) | Lower mortality (25 *vs.* 44 %), incidence of shock, liver failure, bacteremia in "conservative" group; *limitation:* premature termination at 480/660 patients included | 36 |
| IOTA | Meta-analysis / 25 RCT, n=16,037 | General ICU | "Conservative" *vs.* "Liberal", i.e. lower *vs.* higher target according to individual study design | Higher mortality risk (relative risk 1.21 [95%CI 1.0-1.43]) with "liberal" O_2_ strategy (median baseline SpO₂=96% [IQR 96–98%]) | 38 |
| PROSPERO | Meta-analysis + Trial Sequential Analysis / 36 RCT, n=20,166 | General ICU | "Lower" *vs.* "Higher", i.e. lower vs. higher target according to individual study design | No difference in mortality or morbidity | 39 |
| ICU-ROX | Multicenter RCT / n=965 | General ICU; MV | "Conservative" (lowest F_I_O_2_ possible 91≤SpO_2_<97%) *vs.* "Usual" (no limit) | No difference in d28 ventilator-free days and d90/180 mortality | 40 |
| O2-ICU | Multicenter RCT / n=400 | General ICU; expected ICU stay >2 days; ≥ 2 SIRS criteria | Oxygenation target: 8≤PaO_2_≤12 *vs.* 14≤PaO_2_≤18 kPa (≈ 60≤PaO_2_≤90 vs. 105≤PaO_2_≤135 mmHg) | No difference in SOFA score; *limitation:* PaO_2_ < target in "high-normal oxygenation" group | 43 |
| LOCO2 | Multicenter RCT / n=205 | ARDS | "Conservative" (55≤PaO_2_≤70 mmHg, 88≤SpO_2_≤92%) *vs.* "Liberal" (90≤PaO_2_≤105 mmHg, SpO_2_≥96%) until d7 | Premature safety stop for higher mortality in "Conservative" group (d28: 34.3 *vs.* 26.5%; d90: 44.4 *vs.*  30.4%); *limitation:* >50% patients with PaO_2_ > upper level | 63 |
| HOT-ICU | Multicenter RCT / n=2,888 | General ICU; acute hypoxemic respiratory failure | "Lower" (PaO_2_≈60±7.5 mmHg) *vs.* "Higher" (PaO_2_≈90±7.5 mmHg) | No difference in d90 mortality | 64 |
| LUNG SAFE | Sub-study of multicenter, prospective, cohort study /  n=2,005 | ARDS | Presence of d1 "hyperoxemia" PaO_2_>100mmHg), "sustained" (d1 *and* d2) or "excessive" O_2_ (F_I_O_2_≥0.6 + PaO_2_>100mmHg) | 30% hyperoxemia d1, 12% "sustained hyperoxemia", 20% "excessive O_2_" | 65 |
|  | Multicenter retrospective / n=36,307 | General ICU; 24h (3,325 until d5) | PaO_2_<8.9, 8.9≤PaO_2_≤10.6, 12.6≤PaO_2_≤16.4, PaO_2_>16.4kPa (≈ <67, 67≤PaO_2_≤80, 80≤PaO_2_≤95, >95mmHg) | 24h: PaO_2_>16.4kPa (≈ >95mmHg) higher mortality (OR1.23[1.13-1.34]) | 66 |
| IMPACT | Multicenter retrospective / n=16,326 | CPR; ABG within 24h | PaO_2_<60 ("hypoxia"), 60≤PaO_2_<300 ("normoxia"), PaO_2_≥300mmHg ("hyperoxia") | PaO_2_≥300mmHg significantly higher mortality 63(CI:60-66)% *vs.* normoxia 45[CI43-48]%) vs. hypoxia (57[CI56-59]%) | 68 |
|  | Single center, prospective, observational /  n=202 | ARDS | Mean TWA PaO_2_ max. d7 | U-shaped relation mortality/PaO_2_; lowest mortality at 12.5≤PaO_2_≤14 kPa (≈ 94≤PaO_2_≤105 mmHg) | 69 |
|  | Meta-analysis / 33 trials, n=262 | General ICU, healthy volunteers | Mostly F_I_O_2_ 0.21 *vs.*1.0 | Hyperoxia decreases cardiac output; effect most pronounced in heart failure, no effect in sepsis | 72 |
|  | Single center, retrospective / n=503 | General ICU; ICU stay >2 days | PaO_2_>120mmHg | Hyperoxemia associated with VAP incidence; *limitation:* VAP patients more PPI, more shock, need for RBC transfusion, catecholamines, sedation | 73 |
|  | Single center, prospective, observational /  n=93 | General ICU; MV *and* VAP | SpO_2_≥98% during MV | No effect on ICU mortality | 74 |
| HYPER2S | Multicenter RCT / n=442 | Septic shock within first 6 h; MV | F_I_O_2_=1.0 during first 24h *vs.* "standard treatment" | Premature safety stop for higher mortality with "F_I_O_2_=1.0" (d28: 43 *vs.* 35%, p=0.12; d90: 48 vs. 42%, p=0.16); lower number of ventilator-free days (p=0.02), more serious adverse events (p=0.02) despite lower SOFA at d7 (p=0.01) | 75 |
| HYPER2S | Post-hoc analysis of multicenter RCT / n=393 | Septic shock within first 6 h according to Sepsis-3; MV | F_I_O_2_=1.0 during first 24h vs. "standard treatment" | Higher mortality with "F_I_O_2_=1.0" *and* lactate >2mmol/L (d28: 57 *vs.* 44%, p=0.054); d90: 48 vs. 42%, p=0.171); no effect lactate ≤2mmol/L | 76 |
| ICU-ROX | Post-hoc analysis of multicenter RCT / n=251 | Sepsis; MV | "Conservative" (lowest F_I_O_2_ possible 91≤SpO_2_<97%) *vs.* "Usual" (no limit) | Mortality d90 "Conservative" 36.2 *vs.* "Usual" 29.2% (p=0.24); *"…point estimates of treatment effects consistently favored usual O_2_ therapy…"* | 77 |
|  | Multicenter retrospective 7 n=3,422 | Polytrauma with TBI; Mean ISS=21 | 50mmHg PaO_2_-increments; PaO_2_<110 ("hypoxemia"), 110<PaO_2_<487 ("normoxemia"), PaO_2_>487mmHg ("hyperoxemia") | "Extreme" hyperoxemia associated with mortality (OR0.54[CI0.42-0.69], p<0.001) | 85 |
|  | Multicenter, retrospective / n=1,116 | TBI; MV | PaO_2_<10.0kPa),  10.0≤PaO_2_≤13.3kPa (≈ 75≤PaO_2_≤100mmHg) *vs.* PaO_2_>13.3 kPa (≈ >100mmHg) | PaO_2_>13.3 kPa no relation to outcome | 86 |
|  | Multicenter retrospective / n=2,894 | MV; 19% AIS, 32% SAB, 49% ICB | PaO_2_<60, 60≤PaO_2_<300mmHg *vs.* PaO_2_≥300mmHg | PaO_2_≥300mmHg in-hospital mortality 57 vs. 46/47% (p>0.001) | 87 |
|  | Single center retrospective, / n=333 | Thrombolysis for AIS | PaO_2_>120mmHg | PaO_2_>120mmHg: D90 60.6/70.6% cases mRS ≥4 and ≥3 vs. 43.0/56.1% (p<0.01); d90 Mortality 28.6 *vs.* 18.7% (p=0.04) | 88 |
|  | Single center retrospective, / n=197 | SAB | TWA PaO_2_ first 24h and until d6 | Delayed cerebral ischemia *vs.* non-ischemia median PaO_2_ 186(141–213) vs. 161(138–192)mmHg (p= 0.029); poor *vs.* favorable median PaO_2_ 176(154–205) *vs.* 156(136–188)mmHg (p=0.004) | 89 |
|  | Single center retrospective / n=345 | SAB | Highest PaO_2_ within 72h | Vasospasm *vs.* no vasospasm PaO_2_ 232±124 *vs.* 195±101mmHg (p=0.005) | 90 |
|  | Multicenter retrospective / n=432 | SAB; MV | 24h TWA PaO_2_: "low" / "intermediate" / "high" (<97.5 / 97.5≤PaO_2_≤150 / >150mmHg) | TWA-PaO_2_: survivors 118(IQR90-155) *vs.* non-survivors 137(IQR104-167)mmHg (p<001); multivariate analysis no relation TWA-PaO_2_ / outcome | 91 |
| SO_2_S | Multicenter RCT / n=7,635 | AIS | Continuous (2-3L/min) *vs.* nocturnal nasal O_2_ *vs.* control | No difference in mortality and neurological outcome | 92 |
|  | Multicenter, retrospective / n=2,634 | AIS; ICU | Incremental PaO_2_ decile; highest decile PaO_2_>341mmHg | No effect of highest PaO_2_ on mortality | 93 |
|  | Multicenter retrospective / n=24,148 | TBI; MV | PaO_2_ 50mmHg-increments; hyperoxia PaO_2_>300mmHg | No relation PaO_2_ *vs.* mortality except for PaO_2_<60mmHg *and* GCS>12 | 94 |
|  | Multicenter retrospective / n=3,699 | TBI; MV | PaO_2_<60, 60≤PaO_2_<300mmHg *vs.* PaO_2_≥300mmHg | No relation PaO_2_≥300 mmHg *vs.* GOSE<5 at 6 mo | 95 |
| COBRIT | Post-hoc analysis of multicenter RCT / n=417 | TBI, polytrauma; | PaO_2_ 50mmHg-increments within 24h | 6-mo GOSE and cognitive outcome best at 150<PaO_2_<250mmHg | 96 |
|  | Single center retrospective / n=227 | AIS + thrombolysis | 10L/min O_2_ over 4h during thrombolysis | Functional independence 80.8 *vs.* 61.8% thrombolysis alone (p=0.002) | 97 |
|  | Single center RCT / n=68 | TBI, GCS≤8; MV | 6h after trauma F_I_O_2_=0.5 | 6-mo improved GOS (p=0.024), Barthel index and Rankin sclae (both p=0.001); *limitation:* no PaO_2_/SpO_2_ recorded; age>65, co-morbidity, shock excluded | 101 |
| BRAINOXY | Multicenter RCT / n=42 | TBI, GCS≤8; MV | F_I_O_2_=0.7 *vs.* F_I_O_2_=0.4 until max. d14 | No effect on blood markers of oxidative stress, inflammation, neurological injury; *limitation:* age>65 excluded | 102 |
|  | Single center retrospective / n=147 | Polytrauma; prehospital MV | F_I_O_2_=1.0 until ABG analysis within 60min in-hospital | "Severe" (>26.6kPa ≈ >200mmHg) / "Mild" (16<PaO_2_≤26.6kPa≈120<PaO_2_≤200mmHg hyperoxaemia present in 61/20% | 107 |
| VALID | Post-hoc analysis of multicenter RCT / n=472 (TBI n=266) | Polytrauma; ISS=29, MV | Maximum PaO_2_ first 24h | All patients: survivors (PaO_2_ 141[IQR 103-212]mmHg); Non-survivors (148 [IQR105-209]mmHg) (=0.82); Subgroup TBI: PaO_2_133(IQR97-187) *vs.* 152 (IQR108-229)mmHg (p=0.19) | 108 |
|  | Single center retrospective / n=688 | ED; MV, normoxia  (60≤PaO_2_<120mmHg) d1 ICU | Hypoxia/normoxia/hyperoxia PaO_2_<60, 60≤PaO_2_<120, PaO_2_<120mmHg | Hyperoxia present in 43%; mortality 29.7 *vs.* 19.4 (normoxia) and 13.2 (hypoxia) % (p=0.021 vs.normoxia) | 109 |
|  | Multicenter retrospective / n=912 | Polytrauma; median ISS=15; TBI n=1,836, shock n=545) | PaO_2_≥150 *vs.* 60<PaO_2_<150mmHg | Univariate analysis: PaO_2_≥150mmHg mortality 12 *vs*. 9% (p<0.0001); *propensity matching:* OR 0.59(0.50–0.70), p<0.0001 | 110 |
|  | Single center retrospective / n=426 | Blunt chest trauma | First 24h hyperoxia "severe" / "moderate" / "mild" (PaO_2_≥200 / 150≤PaO_2_<200 / 100≤PaO_2_< 200mmHg *vs.* "control" (60≤PaO_2_<100mmHg) | PaO_2_≥200mmHg d28 mortality lower (OR0.23[0.08–0.68]; p<0.001); less VAP, more MV-, iCU-free days | 111 |
|  | Multicenter retrospective / n=855,114 | Polytrauma; median ISS=9 | ED SpO_2_: <94 / 94≤SpO_2_≤97 /  98≤SpO_2_≤100% | Highest ED mortality risk SpO_2_≥98% and/or supplemental O_2_ | 112 |
|  | Post-hoc analysis of multicenter prospective / n=240 | Polytrauma; ISS>15 | PaO_2_≥300mmHg | PaO_2_≥300mmHg less ICU-free days (17[10–21] *vs.* 23[16–26], p<0.001); no effect with MV | 113 |
|  | Multicenter retrospective / n=3,464 | Polytrauma; ICU within 24h | Patient-hours in 90≤SpO_2_≤96 ("normoxia") *vs.* >96% ("hyperoxia"); hyperoxia in 10%- F_I_O_2_ increments until d3 and d4-7 | Increased risk of mortality with higher F_I_O_2_ during hyperoxia | 114 |
| BABICA | Multicenter prospective / n=115 | Out-of-hospital CPR | ABG during CPR / immediately after ROSC | Intra-CPR PaO_2_=85±73mmHg, post-ROSC 128±117mmHg, p=0.046; hospital admission PaO_2_=85±73mmHg *vs.* non-admission (66±39mmHg), p=0.050 | 118 |
|  | Multicenter retrospective / n=145 | Out-of-hospital CPR; ABG during CPR | PaO_2_<60 ("low"), 60≤PaO_2_≤300 ("intermediate"), PaO_2_>300mmHg ("high") | PaO_2_>300mmHg neurologically intact survival 23 vs. 13 / 3% (p=0.062) | 119 |
| IMPACT | Post-hoc of multicenter retrospective / n=4,459 | CPR; ABG within 24h | Highest PaO_2_ 24h ICU | 100mmHg PaO_2_-increments 24% mortality risk increase (OR1.24[CI1.18-1.31]) | 121 |
|  | Single center retrospective / n=170 | CPR; therapeutic hypothermia | Highest PaO_2_ 24h ICU | PaO_2_ significantly related to increased  mortality (p=0.034), poor neurological status (p=0.033) | 122 |
|  | Single center retrospective / n=550 | CPR in-hospital; | First PaO_2_ after ROSC | PaO_2_=146±92 *vs.*171±131mmHg (p=0.65) favorable *vs.* non-favorable neurological outcome; 70≤PaO_2_≤240mmHg associated with favorable neurological function | 123 |
|  | Multicenter prospective / n=280 | CPR; theraoeutic hypothermia | PaO_2_>300mmHg 1,6h post-ROSC | 3% (OR1.03[CI1.02-1.05]) risk increase of poor neurological outcome per 1h hyperoxia duration hour | 124 |
|  | Multicenter retrospective / n=12,108 | CPR; therapeutic hypothermia | PaO_2_≥300mmHg within 24h | PaO_2_≥300mmHg mortality 59(CI56-61)% *vs.* 47(CI45-50% (60≤PaO_2_<300mmHg) / 58(CI57-58)% (PaO_2_<60mmHg) | 125 |
| FINNRESUSCI | Multicenter prospective / n=409 | CPR out-of-hospital | PaO_2_<75 ("low"), 75≤PaO_2_<150 ("middle"), 150≤PaO_2_≤225 ("intermediate"), PaO_2_>225mmHg ("high") | No association hyperoxia vs. neurological outcome | 126 |
|  | Multicenter retrospective / n=544 | CPR; 55% therapeutic hypothermia | PaO_2_>300mmHg 1,6,12,24,48h | PaO_2_≥300mmHg not significantly associated with mortality | 127 |
|  | Multicenter retrospective / n=9,186 | CPR out-of-hospital | PaO_2_≥300mmHg within 24h | PaO_2_≥300mmHg not significantly associated with mortality | 128 |
| TTM | Post-hoc analysis of multicenter RCT / n=869 | CPR out-of-hospital; therapeutic hypothermia | PaO_2_, TWA PaO_2_ 37h post-ROSC; PaO_2_>40 kPa (≈PaO_2_>300mmHg), 8≤PaO_2_≤40 (≈60≤PaO_2_≤300mmHg), PaO_2_<8kPa (≈PaO_2_<60mmHg) | No association with 6-mo neurological outcome | 129 |
|  | Meta-analysis / 7 RCT, n=429 | CPR | "Higher" ("liberal") *vs.* "lower" ("conservative") O_2_ target | Mortality 50 ("liberal") *vs.* 41% ("conservative", p=0.04) | 130 |
| COMACARE | Multicenter RCT / n=120 | CPR out-of-hospital | 36h ICU "Moderate hyperoxia" (20≤PaO_2_≤25kPa ≈ 150≤PaO_2_≤188mmHg) *vs.* "normoxia" (10≤PaO_2_≤15kPa ≈ 75≤PaO_2_≤113mmHg) | No effect on neuron-specific enolase, S100β | 131 |
| ICU-ROX | Post-hoc analysis of multicenter RCT / n=166 | "*Suspected hypoxic ischaemic*  *encephalopathy*"; MV | "Conservative" (lowest F_I_O_2_ possible 91≤SpO_2_<97%) *vs.* "Usual" (no limit) | D180: mortality "conservative" 43 vs. "usual" 59% (p=0.15); "*unfavourable neurological outcome*" "conservative" 55 *vs.* "usual" 68% (p=0.15) | 132 |
| DETO2X-SWEDEHEART | Multicenter RCT / n=6629 | AMI | 6L/minO_2_ 6-12h | No effect on 1-year outcome | 138 |
| AVOID | Multicenter RCT / n=441 | STEMI | 8L/minO_2_ until ward | 8L/minO_2_ more recurrent MI (5.5 vs. 0.9%, p=0.006), higher infarct size (p=0.04); *limitation:* lower mortality (1.8 vs. 4.5%, p=0.11) | 139 |
| Oxygen Therapy in Acute Coronary Syndromes | Multicenter crossover RCT / n=40,872 | ACS | 6-8L/minO_2_ *vs.* 90≤SpO_2_<95% | No effect on d30-mortality | 140 |
|  | Multicenter RCT / n=500 | Elective open colorectal resection | F_I_O_2_=0.8 *vs.* 0.3 until 2h post-op | F_I_O_2_=0.8 5.2 *vs.* 11.2% (p=0.01) | 142 |
| PROXI | Multicenter RCT / n=1,386 | Elective / acute laparotomoy | F_I_O_2_=0.8 *vs.* 0.3 until 2h post-op | F_I_O_2_=0.8 19.1 *vs.* 20.1% (p=0.64) | 143 |
| Supplemental Oxygen in Colorectal Surgery | Single center prospective / n=5,749 | Major intestinal surgery >2h | F_I_O_2_=0.8 *vs.* 0.3 every 2 weeks alternating intervention study | 30d-SSI F_I_O_2_=0.8 10.8 *vs.* 11.0% (p=0.85) | 144 |
| PROXI | Post-hoc analysis of multicenter RCT / n=1,382 | Elective / acute laparotomoy | F_I_O_2_=0.8 *vs.* 0.3 until 2h post-op | F_I_O_2_=0.8 2.3-year mortality 23.2 *vs.* 18.3% (p=0.03); difference due to patients with cancer surgery | 145 |
| PROXI | Post-hoc analysis of multicenter RCT / n=1,377 | Elective / acute laparotomoy | F_I_O_2_=0.8 *vs.* 0.3 until 2h post-op | F_I_O_2_=0.8 3.9-year cancer-free survival significantly shorter, long-term mortality significantly higher vs. F_I_O_2_=0.3 (p=0.04) | 146 |
| Intra-operative Inspiratory Oxygen Fraction and Postoperative Respiratory Complications | Multicenter retrospective / n=79,322 | General surgery | Quintiles F_I_O_2_=0.31,0.41,0.52,0.79 | Dose-dependent association F_I_O_2_ *vs.* d7-"Major respiratory complications composite"; d30-mortality. | 151 |
|  | WHO Meta-analysis / 1/ RCT, 2 other trials | General surgery | F_I_O_2_=0.8 *vs.* 0.30-0.35 | F_I_O_2_=0.8 "no signal of harm" *vs.* 0.30-0.35 | 152 |
|  | WHO Meta-analysis / 12 RCT, n=5,976 | General surgery | F_I_O_2_=0.8 *vs.* 0.30-0.35 | F_I_O_2_=0.8 reduces SSI risk *vs.* 0.30-0.35 (OR0.80[CI0.64-0.99], p=0.043): general anaesthesia with tracheal intubation. | 153 |
|  | Single center RCT / n=210 | Open surgery for appendicitis | F_I_O_2_=0.8 *vs.* 0.30 until 2h post-op | F_I_O_2_=0.8 SSI 5.6 *vs.*13.6% (p=0.04); hospital stay 2.51 vs. 2.92 (p=0.01) | 156 |
| Cochrane Perioperative Oxygen Review | Meta-analysis / 10 RCT, n=1,458 | General surgery | "Higher" vs. "lower" F_I_O_2_ | "Higher" vs. "lower" F_I_O_2_ "very low evidence" serious adverse event risk | 157 |
|  | Meta-analysis / 12 trials, n=28,984 | General ICU; MV | F_I_O_2_ "low" *vs.* "high" (as defined  by authors) | F_I_O_2_ "high"; no impact on pneumonia, ARDS, MV duration; F_I_O_2_≥0.8 increased risk of: atelectasis | 158 |
| ICU-ROX | Post-hoc analysis of multicenter RCT / n=125 | General ICU; MV | "Conservative" (lowest F_I_O_2_ possible 91≤SpO_2_<97%) *vs.* "Usual" (no limit) | No effect on blood biomarkers of oxidative stress | 161 |
